# Novel Preparation of Monodisperse Microbubbles by Integrating Oscillating Electric Fields with Microfluidics

**DOI:** 10.3390/mi9100497

**Published:** 2018-09-27

**Authors:** Anjana Kothandaraman, Anthony Harker, Yiannis Ventikos, Mohan Edirisinghe

**Affiliations:** 1 Department of Mechanical Engineering, University College London, London WC1E 7JE, UK; A.Kothandaraman@bham.ac.uk (A.K.); y.ventikos@ucl.ac.uk (Y.V.); 2 Department of Physics and Astronomy, University College London, London WC1E 6BT, UK; a.harker@ucl.ac.uk

**Keywords:** microfluidics, superimposed electric fields, microbubbles, CFD

## Abstract

Microbubbles generated by microfluidic techniques have gained substantial interest in various industries such as cosmetics, food engineering, and the biomedical field. The microfluidic T-junction provides exquisite control over processing parameters, however, it relies on pressure driven flows only; therefore, bubble size variation is limited especially for viscous solutions. A novel set-up to superimpose an alternating current (AC) oscillation onto a direct current (DC) field is invented in this work, capitalising on the possibility to excite bubble resonance phenomenon and properties, and introducing relevant parameters such as frequency, AC voltage, and waveform to further control bubble size. A capillary embedded T-junction microfluidic device fitted with a stainless-steel capillary was utilised for microbubble formation. Furthermore, a numerical model of the T-junction was developed by integrating the volume of fluid (VOF) method with the electric module; simulation results were attained for the formation of the microbubbles with a particular focus on the flow fields along the detachment of the emerging bubble. Two main types of experiments were conducted in this framework: the first was to test the effect of applied AC voltage magnitude and the second was to vary the applied frequency. Experimental results indicated that higher frequencies have a pronounced effect on the bubble diameter within the 100 Hz and 2.2 kHz range, whereas elevated AC voltages tend to promote bubble elongation and growth. Computational results suggest there is a uniform velocity field distribution along the bubble upon application of a superimposed field and that microbubble detachment is facilitated by the recirculation of the dispersed phase. Furthermore, an ideal range of parameters exists to tailor monodisperse bubble size for specific applications.

## 1. Introduction

Gas encapsulated microbubbles stabilised by a surfactant or polymer coating have gained substantial interest among researchers over the past two decades, branching out into various applications such as food engineering, water purification, and biomedical engineering. For example, microbubbles have also been shown to enhance the digestibility, flavour intensity, as well as the shelf life of products [1,2,3]. Additionally, they have been used as texture modifiers and as nutraceutical carriers to develop novel functional foods that may have physiological benefits [4]. Contrasting applications include waste water and sewage treatment [5], in particular ozonation [6] and floatation [7], which incorporate bubbles <150 µm to separate particulates from potable water by harnessing the high surface area to volume ratio, which improves the mass transfer rate [4]. Microbubbles play a vital role in the preparation of contrast agents for ultrasound imaging. Their application extends beyond contrast agents into sonoporation, tumour ablation, sonothrombolysis, and as a carrier of genes and drugs [8,9,10]. Over the years, various methods have been utilised for the preparation of microbubbles. Mechanical agitation and sonication [11,12] are two of the most extensively reported methods. Wide bubble size distributions were recorded in these methods, which would require additional fractioning techniques [13] to eradicate larger bubbles and retain monodispersity, which is a crucial factor in diagnostic and therapeutic applications. More recently, co-axial electrohydrodynamic atomisation (CEHDA) [14] was used to prepare microbubbles. Although CEHDA achieves a narrower size distribution in comparison with aforementioned techniques; polydispersity can still be ~30–40% [14].

Microfluidics, on the other hand, have established their versatility in various fields for effective microbubble generation and novel pressurised gyration [12]. Peyman et al. [15] demonstrated a flow-focusing device to prepare microbubbles for specifically clinical applications. Their on-chip device was fabricated using photolithography and consisted of very fine channel diameters (30–50 µm) and required very low liquid flow rates and gas pressures of 20–50 μL min^−1^ and 10–40 psi, respectively. Similarly, Castro-Hernandez et al. [16] developed a flow-focusing device with channel diameters of 50 μm, however, they required the use of less viscous liquids. On the other hand, the use of a T-junction device has also been widely investigated because of the exquisite control they offer over processing parameters such as solution flow rate and gas pressure, which govern the dynamics of droplet formation and detachment in the junction. This device also has the benefits of low set-up costs, natural scalability, and the convenience to replace capillaries in the event of blockage. Fluid flow in the capillaries is controlled by high precision mechanical pumps and gas pressure is controlled by adjusting the regulator on a gas tank. An ideal range and combination of parameters exists to attain the desired microbubble features. At a constant liquid flow rate, minimum and maximum inlet gas pressures can be defined as g_min_ and g_max_, respectively [17]. Any pressure below g_min_ would cause the liquid stream to push the gas stream back up the capillary as a result of the capillary force of the liquid. Increasing the pressure past g_max_ disturbs the overall laminar flow of both streams in the outlet capillary and pushes the liquid stream back into the liquid inlet capillary. Bubble sizes between 65 µm and 170 µm can be achieved by simply varying these processing parameters; however, without an external force, bubble size modulation in microfluidic techniques is limited under this small window of parameter variation, especially when viscous solutions are used.

The desirable target is to obtain optimal control over the production of the microbubbles and to tailor their size to suit the diverse range of applications. The effect of direct current (DC) electric fields on bubbles has engaged the interest of various researchers [18,19]. Di Marco et al. [18] explored the electrohydrodynamic effect on nitrogen bubbles. They noted that the detachment of bubbles did not occur at low liquid flow rates. On the other hand, the presence of a high voltage electric field (5–15 kV) not only promoted bubble detachment, but generated a force that effectively moved the bubbles away from the inlet orifice. Sunder and Tomar [19] simulated the effect of various electrical stresses applied tangentially at the tip of a needle. Their studies indicated that non-uniform electric stresses enable effective control of the frequency and volume of the bubbles produced. This is because localised electric stresses at the bubble interface result in premature neck formation and eventually detachment; therefore, varying the potential difference applied at the nozzle can allow modification of the bubble volume. For this reason, the idea of merging electrohydrodynamics into the set-up was explored by introducing a DC electric field at the outlet capillary [20]. This introduces an electric field perpendicular to the leading edge of the droplet. This dominant tangential force overpowers the interfacial tension and shear forces. Radial acceleration in the axial direction facilitates the break-up of bubbles and a distinct reduction of bubble size was observed by these researchers [20]. However, they observed that microbubble size reduction ceased at voltages above 12 kV.

Another idea that has been explored in literature is the superimposition of an alternating current (AC) on a DC electric field [21,22,23] to generate liquid droplets by electrospraying. Jaworek et al. [21] indicated that this divides the waveform into two components—the AC and the DC. The DC component is applied by shifting the zero line up to a specific voltage, which acts as the new time base. The AC component is introduced as a waveform oscillating about this new time base at a peak-to-peak (P–P) voltage. For instance, setting the DC voltage to 5 kV with a superimposed AC voltage of 4 kV P–P oscillates the waveform between 3 kV and 7 kV. Depending on the applied frequency and amplitude, the droplets undergo non-linear oscillations, which then results in their break-up [24]. Balachandran et al. [22] superimposed AC on DC electric field to facilitate liquid droplet formation in their electrospraying apparatus. They noticed that increasing the frequency of the applied signal resulted in an increase in droplet formation rate and a subsequent decrease in droplet size, suggesting that frequency is an important contributor to droplet break-up.

Movassat et al. [24] modelled the oscillatory behaviour of a bubble when subjected to large vibrations. They reported that a bubble begins to deform, resulting in the formation of a dip in the centre; when this oscillatory force increases, the bubble undergoes break up. In terms of bubble dynamics, the resistance to change in velocity (inertia) of the liquid penetrates the centre of the bubble, altering the bubble volume [25], until the liquid penetrates the entire sphere breaking it up into smaller bubbles. Jagannathan et al. [26] observed rapid fragmentation of bubbles at high frequencies as a result of the cavitation and subsequent deformation of the larger bubbles, which is in accordance with the research conducted by Movassat et al. [24]. Although the behaviour of a bubble subjected to oscillating fields has been investigated, mass preparation of bubbles has not been reported using this method.

Various experimental configurations have been employed to introduce an oscillating electric field to an electrospraying set-up; however, to the best of the authors’ knowledge, oscillating electric fields have not been integrated into microfluidic arrangements. In this work, we present a novel apparatus to superimpose an AC on DC electric field to a microfluidic system. We investigate the effect of superimposed AC and applied frequency on bubble formation. We show that high AC voltage increases the bubble size, probably because of heating effects, whereas subjecting a bubble to high frequencies close to its resonance frequency could accelerate bubble break-up.

## 2. Theoretical Aspects

### Scaling Models Associated with Bubble Formation

Prior to discussing the effects of the electric field on the formation of bubbles, it is essential to discuss the scaling models that have been associated with the formation of bubbles. Various studies have been conducted to establish a better understanding of bubble and droplet formation within microfluidic devices [27,28,29,30]. The very first experiments using two phase microfluidic systems were conducted by Thorsen et al. [27], where the droplet dynamics for a classic oil-in-water system was discussed. The discontinuous phase (water) blocks the continuous stream (oil), causing an increase in shear forces at the leading edge of emerging water droplet.

The flow is described to be non-linear as the boundary is not static as the movement of one fluid will consequently affect the other. This results in the formation of an instability, which facilitates the formation of droplets, owing to the competition between surface tension and shear forces, which was detailed in depth in the work of Taylor [28]. This work implies that the movement of a secondary fluid causes the distortion of a droplet due to the presence of tangential viscous and dynamical forces acting on the droplet surface, the surface tension retains the spherical shape of the emerging droplet.

Garstecki et al. [29] investigated the dynamics of the break-up mechanism of the droplet. They noticed that past a critical value and when the interfacial tension exceeds the shear stress, a pressure drop is generated across the bubble. This pressure drop arises as the bubble blocks the entire width of the capillary, resulting in an increase in pressure upstream, leading to the thinning of the droplet neck. This process is referred to as the ‘squeezing’ regime, for which these authors developed a scaling model for the resultant droplets formed:

(1) $$\frac{l}{w}=1+\alpha \frac{Qx}{Qy}$$

where *l* is the length of the droplet; *w* is the width of the channel; *Qx/Qy* are the rates of flow of the dispersed and carrier fluids, respectively; and α is constant of order one, whose value depends entirely on the geometry of the T-junction in use. To add to this theory, De Menech et al. [30] presented the ‘dripping’ regime to describe the mechanism of break-up of the emerging droplet. It was shown that this process of break-up occurs at higher values of the Capillary Number (*Ca* ≥ 0.02).

(2) $$Ca=\frac{\mu V_{l}}{\gamma }$$

where *µ* is the viscosity, *V*_l_ is the mean velocity of the continuous medium, and *γ* is the interfacial tension. As the expanding globe fills up the entire capillary, only a thin lubricating film separates the bubble from the channel walls. The continuous fluid, which is still at a constant flow rate, begins to seep through this film. As a result of a reduction in surface area, velocity (*V*_l_) consequently increases, resulting in a higher capillary number. This causes an increase in shear stress between the thin film and the bubble, which pushes it further in the axial direction, eventually causing the neck to disjoin from the fluid stream. The stream periodically retracts back to the inlet orifice.

## 3. Materials and Methods

### 3.1. Solution

Glycerol of 99% purity, purchased from Sigma Aldrich, UK, was diluted with distilled water. PEG-40-S (polyethylene glycol-40-stereate, Sigma, Aldrich, UK) is a non-ionic/neutral surfactant. It was added to the solution to reduce surface tension and facilitate bubble formation. The physical properties of the solution used to prepare bubbles are shown in Table 1.

### 3.2. Characterisation of Solution

The density of the solutions was measured using a 25 mL DIN ISO 3507 Gay–Lussac type density bottle (VWR International, Lutterworth, UK). The viscosity was assessed using an Ostwald Viscometer (Schott Instruments GmbH, Mainz, Germany). The surface tension of the solution was measured using a Wilhelmy Plate tensiometer (Kruss, Hamburg, Germany) and the electrical conductivity was studied using a Jenway 3540 conductivity meter (Bibby Scientific, Stone, UK). Equipment used was calibrated prior to use.

### 3.3. Experimental Setup

**Figure 1:**
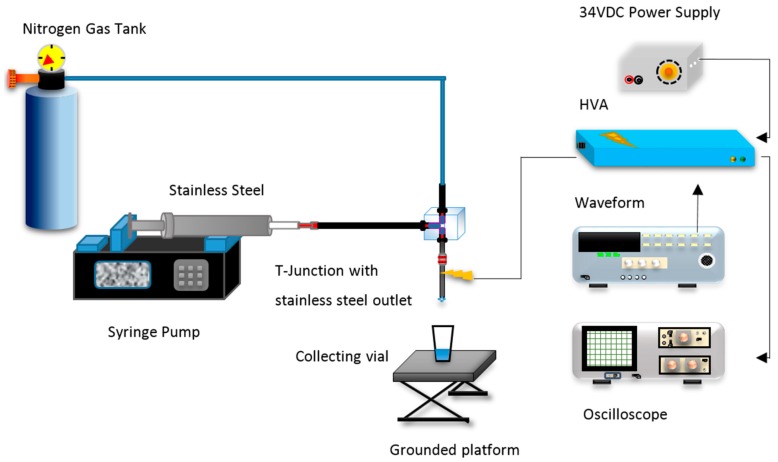
Schematic of experimental set-up used to superimpose alternating current (AC) onto a direct current (DC) electric field. HVA—high voltage amplifier.

The purpose of this experimental set-up (Figure 1) is to superimpose an AC field onto a specified DC field [31]. Waveform generators allow this by applying an offset to the output signal. This resultant signal, ranging from 0–10 V, is used to drive the input of the programmable high voltage amplifier (HVA) (Ultravolt, New York, NY, USA) which amplifies 0–10 V to 0–20 kV. The output signal can be viewed using an oscilloscope by connecting it to the appropriate output pin. The amplifier has a 15-pin D-type female socket, which enables the complete programming of the unit. A schematic of the pin arrangement is given in Supplementary Information 1 (SI 1).

### 3.4. Bubble Generation

Two fluorinated ethylene propylene tubes of 100 µm internal diameter were connected to the two inlets of the T-junction, as shown in SI 1. A stainless-steel capillary of the same dimensions was connected to the outlet to provide a conductive surface to apply the electric field. The top inlet was connected to a pressurised gas tank containing nitrogen, and the second inlet was connected to a stainless-steel syringe that supplies the solution at a constant flow rate. The fluid was injected at a constant flowrate of 100 µL/min, with a constant pressure of 256 kPa. These two fluids meet at the junction area, where bubbles form, which traverse through the outlet capillary and are collected on a glass slide.

All experiments were conducted under an ambient temperature of 22 °C and a relative humidity of 37%. In this work, there was no specific attempt to study bubble stability as only a simple glycerol-water model system was used, however, the monodisperse microbubbles prepared were stable for at least 60–90 min. High speed camera videos of the bubble formation under various applied electric field scenarios are given in SI 2–5.

### 3.5. Characterisation of Bubbles

Microbubbles were collected on a glass slide and observed under an optical microscope (Nikon Co, Tokyo, Japan) immediately after generation. The bubbles were observed at ×5, ×10, and ×20 magnification. The bubble formation is exemplified in the high-speed camera videos (Photron SA1.1, full frame resolution of 1024 × 1024 pixels) under various applied electric field scenarios (SI 2–5). The measurements were carried out using Image J digital imaging software (ImageJ 1.46r, National Institute of Health, Bethesda, MD, USA). The diameters of 50 bubbles were measured from the images captured for analysis.

### 3.6. Computational Modelling Methodology

The accuracy and reliability of experimental investigations can be further improved if the dynamics of the flow fields can be observed in greater detail. This is achievable because of progress in multiphase/multiphysics computational fluid dynamics (CFD).

A numerical model to study the hydrodynamic and electrohydrodynamic interactions was created using the Multiphysics suite CFD-ACE + (ESI group, Paris, France). A structured 2D grid consisting of the two inlet channels that pump the liquid and gas into the junction and a stainless-steel outlet capillary where charge is applied and where the formed bubbles traverse constitute the main components of this model.

### 3.7. Modelling the Hydrodynamics

The incompressible mass and momentum conservation laws are discretised on a structured mesh using the finite volume method (FVM). The simulation of hydrodynamic fluid flow is based on the iterative solution of this discretised set of equations:

(3) ∇.u=0

(4) $$\rho \left(\frac{du}{dt}+u\times \nabla u\right)=-\nabla p+\nabla \times \left(µ\left(\nabla u+\nabla u^{T}\right)\right)+F^{s}+F^{el}$$

where *u* is the fluid velocity, *p* is the fluid pressure, *ρ* is the fluid density, and *µ* is the dynamic viscosity of the fluid. *F^s^* and *F^el^* are the surface tension and electric tensors, respectively.

The surface tension is based on the Young–Laplace equation and is expressed by Equation 5 in terms of the surface curvature [32].

(5) $$F^{s}=\sigma kň$$

where *k* is the surface curvature, σ is the surface tension coefficient, and ň is the surface normal. The continuum surface force (CSF) model developed by Brackbill et al. [33] has been integrated in various available commercial codes [34], it converts the interfacial surface into a volume force such that it can be added as a source term in Equation (4). The surface tension force is implemented by the piecewise-linear interface calculation (PLIC) scheme, which enables the accurate calculation of the curvature of the droplets that are formed for reconstruction of the interface front [35].

The gas and liquid phases were resolved within the domain using the volume of fluid (VOF) model. In this algorithm, a scalar parameter *f* represents a volume fraction for the liquid inside a cell. The volume fraction *f* describes the phase distribution across each control volume: when *f* = 1, it indicates that the cell is completely filled by liquid and *f* = 0 represents a cell filled with gas. Any other state has a value of 0 < *f* < 1 and indicates that the interface traverses the cell. The time propagation of the volume fraction can be calculated by resolving the passive transport equation.

(6) $$\frac{df}{dt}+\nabla \times \left(uf\right)=0$$

The conservation of mass is satisfied intrinsically and topological variations experienced at the interface are handled naturally without compromising the precision [36], thus deeming the VOF a viable method for simulating the formation of microbubbles under the influence of electric fields.

### 3.8. Modelling the Electrohydrodynamics

In addition to the hydrodynamics of the system, the electrohydrodynamic interactions as a result of the superimposed electric field were modelled by the application of the electric module of the CFD-ACE + suite. The module facilitates the calculation of various electric quantities based on the solution of the Maxwell’s equations. The electric momentum (*F^el^*)) is defined by Maxwell’s stress tensor [37], by the following expression:

(7) $$F^{el}=q\vec{E}-\frac{1}{2}\vec{E}\times \vec{E}\nabla \varepsilon +\frac{1}{2}\nabla \left(\rho \frac{\delta \varepsilon }{\delta \rho_{T}}{\vec{E}}^{2}\right)$$

In this equation, q represents the volume charge density at the interface, $\vec{E}$ is the electric field vector, and *ε* is the permittivity of the fluid. The first term in Equation (7) denotes the coulombic force, which occurs as a result of the interaction between the charged interface and the electric field. The second and third terms embody the polarisation forces and are referred to as the dielectric and the electrostrictive forces, respectively. For a homogenous and incompressible fluid, the permittivity has no gradient and the dielectric force equals zero. For the present problem, it is adequate to consider the electrohydrodynamic effects within the electrostatic limit only. This is because the current output at the load is very low and occurs over a prolonged period of time, thus the electromagnetic effects can be neglected. The electrostrictive force can be neglected in this case because of the incompressible nature of the fluids used in this work. Therefore, Equation (7) can be further simplified as follows:

(8) $$F^{el}=q\vec{E}-\frac{1}{2}\vec{E}\times \vec{E}\nabla \varepsilon$$

The governing equations for the electric field need to be resolved in order to obtain the Maxwell stress terms. Based on Gauss’s Law, applying an electric potential generates an electric field, this law asserts that the flux of that field passing through any closed surface is proportional to the total charge contained within that surface [38] and can be expressed in differential form as shown below.

(9) ∇×D=q

where *D* is the electric displacement flux density, given in (C/m^2^). The flux density *D* can be correlated with the electric field E by the following expression.

(10) D= εE

where electric permittivity can be expanded by the following equation [39]:

(11) $$\varepsilon =\varepsilon_{r}\varepsilon_{0}$$

where *ε_r_* is the dielectric constant and *ε*_0_ is the permittivity of vacuum. The electric field vector *E* is non-rotational in the absence of time-varying magnetic fields, that is, ∇×E=0. Therefore, a scalar electric potential (*Ψ*) can be defined by the following expression:

(12) E=−∇Ψ

Substituting Equations (10) and (12) into Equation (9), we deduce the Poisson’s equation for the electric potential denoted by Equation (13).

(13) $$\nabla \times \left(\varepsilon_{r}\varepsilon_{0}\nabla \Psi \right)=-q$$

Using Equation (13), the electric potential distribution within the system under different electric fields can be calculated. Lima et al. [40] added that the characteristic time for electrostatic processes is large in contrast to magnetic phenomenon; therefore, the aforementioned electrostatic equations sufficiently describe an electrohydrodynamic system. The heating effects are not modelled as part of this work.

## 4. Results and Discussion

### 4.1. Effect of Superimposed AC

Superimposing an AC field onto a DC one introduces three new parameters into the system—waveform type, AC P–P voltage, and frequency. The effect of these parameters on the formation of microbubbles was investigated to understand their effect on bubble diameter. The first series of experiments was conducted by testing two different applied AC voltages—2 kV P–P and 4 kV P–P. For 2 kV P–P, the waveform oscillates 1 kV either side of the zero marker, and for 4 kV P–P, the wave oscillates 2 kV from 0 to peak, and from 0 to trough.

**Figure 2:**
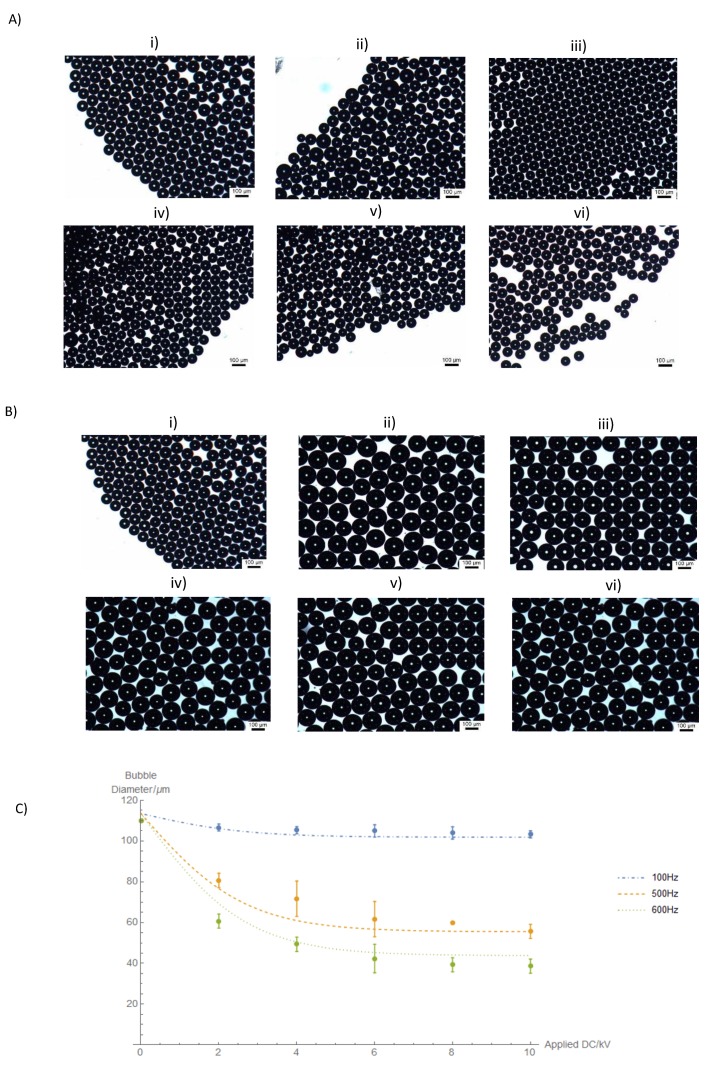
(**A**) Optical micrographs of bubbles obtained at a superimposed AC voltage of 2 kV P–P at DC voltages of (**i**) 0 V, (**ii**) 2 kV, (**iii**) 4 kV, (**iv**) 6 kV, (**v**) 8 kV, and (**vi**) 10 kV. (**B**) Optical micrographs of microbubbles obtained at a superimposed AC voltage of 4 kV P–P at DC voltages of (**i**) 0 kV, (**ii**) 2 kV, (**iii**) 4 kV, (**iv**) 6 kV, (**v**) 8 kV, and (**vi**) 10 kV. (**C**) Variation of microbubble diameter with applied DC voltage at different P–P AC voltages, error bars represent three standard deviations, estimated from repeat experiments. Curves represent a fit to a simple analytic function.

For these tests, the waveform used was a sinusoidal wave with a frequency of 500 Hz. The applied AC was modulated by varying the applied P–P voltage on the waveform generator. AC voltages of 2 kV P–P, 3 kV P–P, and 4 kV P–P were investigated. Once the P–P AC was set, the DC voltage was increased from 2 kV up to 10 kV by increasing the DC offset. Figure 2 shows optical micrographs of microbubbles obtained by progressively increasing the applied DC voltage. It can be seen that increasing the DC voltage at this superimposed AC voltage causes a drop in bubble diameter. The average bubble diameter without an application of an electric field was ~110 µm, when a DC voltage of 2 kV was applied, the average bubble diameter dropped to ~80 µm. Increasing the applied DC voltage to 10 kV further reduces the bubble diameter to ~55 µm, however, increasing the voltage past 10 kV increased the polydispersity of the bubbles due to ionization of the surrounding air; similar behaviour was observed by Parhizkar et al. [20] at 12 kV.

On the other hand, increasing the AC voltage to 4 kV (i.e, for an applied DC voltage of 8 kV, the waveform will oscillate between 6 kV and 10 kV) had the opposite effect on the bubble diameter. From Figure 2C, it can be seen that the relationship between the bubble diameter and applied AC voltage is directly proportional, this could be because of the fact that prolonged exposure of the bubble to high AC fields combined with high frequency can result in the superheating of the bubble as reported by Cheng and Chaddock [41]. In their work, they mentioned that bubbles tend to grow under ambient conditions as a result of evaporation at the gas–liquid interface. Spheroidal structures tend to have a higher surface area than spherical ones, hence heat is effectively conducted across the boundary of the bubble.

Quan et al. [42] added that deformation of any kind, coalescence, or bursting are caused by disturbances on the liquid/vapour boundary layer induced by the bubble oscillation caused by high AC fields. Kweon et al. [43] observed the effect of high voltage AC fields on the bubble dynamics, rapid oscillations and subsequent droplet detachment was observed. They revealed that these rapid oscillations at high applied AC fields promoted electrohydrodynamic convection across the bubble surface.

Heat generated by high AC fields may have also contributed to heating effects, which increase the overall bubble growth time. Gao et al. [44] verified that at a higher flux, the amount of time for the bubble to expand increases at the same applied voltage. In this work, it can be observed that lower AC voltages are optimal for the bubble formation as the overall bubble growth time is decreased. The effect of superimposed AC is summarised in Figure 2C, suggesting that a lower superimposed AC voltage of 2 kV P–P is optimal for this work.

### 4.2. Effect of Frequency

**Figure 3:**
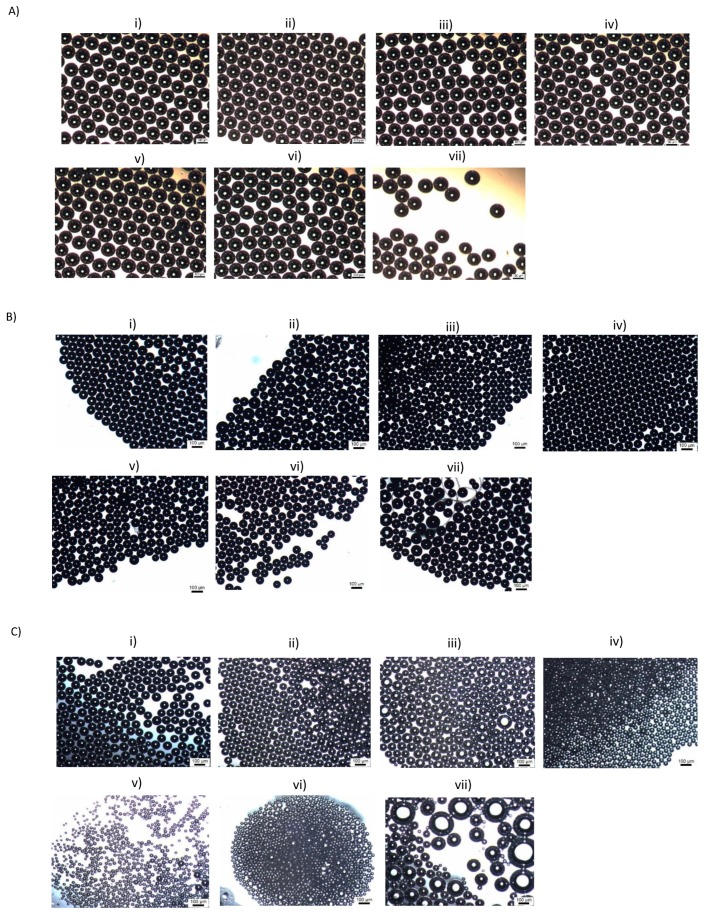
(**A**) Optical micrographs of bubbles obtained at a superimposed AC voltage of 2kV P–P, 100 Hz at applied DC voltages of (**i**) 0 V, (**ii**) 2 kV, (**iii**) 4 kV, (**iv**) 6 kV, (**v**) 8 kV, (**vi**) 10 kV, and (**vii**) 12 kV. (**B**) Optical micrographs of bubbles obtained at a superimposed AC voltage of 2kV P–P, 500 Hz at applied DC voltages of (**i**) 0 V, (**ii**) 2 kV, (**iii**) 4 kV, (**iv**) 6 kV, (**v**) 8 kV, (**vi**) 10 kV, and (**vii**) 12 kV. (**C**) Optical micrographs of bubbles obtained at a superimposed AC voltage of 2 kV P–P, 600 Hz at applied DC voltages of (**i**) 0 V, (**ii**) 2 kV, (**iii**) 4 kV, (**iv**) 6 kV, (**v**) 8 kV, (**vi**) 10 kV, and (**vii**) 12 kV.

Figure 3 shows typical microbubbles obtained at a specific frequency at a constant superimposed AC voltage. Frequencies of 100 Hz, 500 Hz, and 600 Hz were tested. Based on the results acquired for the effect of superimposed AC voltage on bubble diameter, an optimal AC voltage of 2 kV P–P was maintained for all the experiments. When a low frequency of 100 Hz was utilised, the bubble diameter dropped from 110 µm to 100 ± 0.60 µm as the applied DC voltage was increased in steps of 2 kV up till 12 kV.

Increasing the applied frequency to 500 Hz resulted in a drop in bubble diameter from 110 µm to 55 ± 1.2 µm when the applied DC voltage was increased from 0–12 kV in steps of 2 kV. Microbubbles acquired at 6 kV (Figure 3(Biv)) displayed near-perfect monodispersity. Application of 12 kV had a negative effect on the monodispersity and stability of the microbubbles as shown in Figure 3(Bvii).

**Figure 4:**
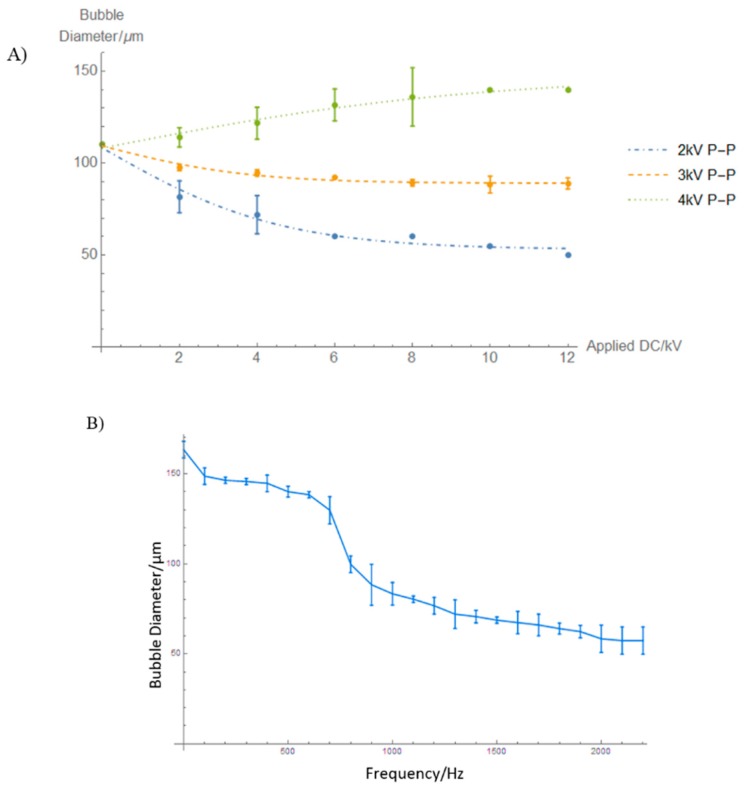
(**A**) Effect of applied AC electric field frequencies on the microbubble diameter. Error bars represent three standard deviations, estimated from repeat experiments. Curves represent a fit to a simple analytic function. (**B**) Variation of microbubble diameter as a function of frequency at 2 kV P–P AC voltage, 6 kV DC Voltage, 50 bubbles were used for the measurements, error bars represent three standard deviations, estimated from three repeat experiments. Measured points are connected by straight line segments as a guide to the eye.

Increasing the frequency to 600 Hz displayed interesting results, at an applied DC voltage of 8 kV (Figure 3(Cv)), the bubble size was recorded to 38 ± 1.2 µm, but increasing the voltage further past this point resulted in a foam-like cluster, as shown in Figure 3(Cvi). This coalescence is likely to be because of the effects of heating due to presence of high electric fields, as pointed out by Gao et al. [44]. From the results presented above, frequency is a key parameter in these studies. As the frequency is increased, there is a clear and significant decrease in microbubble diameter by 50% (Figure 4A). The polydispersity index of the microbubbles produced using this method was calculated, and ranged between 0.3–4%, which is significantly lower than what was observed by Farook et al. [14].

From Figure 4B, it can be observed that the applied frequency results in the decline of microbubble diameter as it is increased to 2.2 kHz. A microbubble diameter reduction of 170 to 138 ± 0.6 μm was observed between 100 and 600 Hz. A decline in bubble size from 138 ± 0.6 µm to 99 ± 1.5 μm was observed between 600 and 900 Hz and 99 ± 1.5 to 57 ± 2.5 μm was observed at 800–2200 Hz. These results are indicative of a complex system that is subjected to various parameters that are non-linear in nature. The sudden drop in bubble diameter observed in Figure 4B between 600 and 900 Hz can possibly be attributed to the resonance of the bubble of that diameter as a result of the applied frequency. Kweon et al. [43] also observed a sudden reduction in the bubble size and referred to this as the critical AC voltage in their study. They added that the critical voltage occurs at lower applied voltages as the electrode gap decreases and the needle height increases. They concluded that the critical voltage also depends on the configuration of the nozzle. When in the presence of a sound field, a bubble has been shown to vibrate or resonate non-linearly. For example, in order to reduce the bubble to size of 2 µm, a frequency of the order of 10 MHz may be required [45]. This is very similar to the resonance frequency of a microbubble of that size. The resonance frequency is given by Minnaerts’ theory [46]:

(14) $$f_{M}=\frac{1}{R_{0}2\pi }\sqrt{\frac{3\gamma p_{0}}{\rho }}$$

where $f_{M}$ is the Minnaert resonance frequency, *ρ* the density of surrounding liquid, γ is the polytrophic constant, *р*_0_ is the hydrostatic pressure outside the bubble, and *R*_0_ is the equilibrium bubble radius. Based on Minnaert’s theory for bubble resonance, the resonance frequencies of the various microbubble diameters acquired in the experiments were calculated. The results indicate that the frequencies required to resonate smaller bubbles are higher than the frequencies used in this work, as anticipated. However, this equation omits other variables that contribute to the microbubble formation process such as surface tension, electric stresses from the AC/DC components, and solution properties. Plesset and Prosperetti [47] developed a more accurate expression taking to consideration the effects of surface tension:

(15) $$f_{0}^{2}=\frac{1}{{\left(2\pi \right)}^{2}}\left[3\gamma \frac{p_{0}}{\rho R_{0}^{2}}-\frac{2\sigma }{\rho R_{0}^{3}}\right]$$

where $f_{0}$ the resonance frequency, *р*_0_ is the equilibrium pressure, and *σ* is the surface tension. Substituting an $R_{0}$ value of 25 μm into Equation (15), we obtain a frequency value of 124.3 kHz, that is, according to this expression, a frequency of 124 kHz is required to resonate a microbubble with a diameter of 50 μm. However, using the experimental set-up described in this manuscript, a frequency of 1200 Hz combined with 2 kV P–P AC and 6 kV DC is adequate to reduce a microbubble with a diameter of ~80 μm to ~70 μm. Using the Plesset-Prosperetti equation minimised the discrepancy slightly as the surface tension effects were taken to account, although still neglecting the electric stresses and the solution properties. It must also be noted that this expression correlates the resonance frequency and radius for a stationary bubble in a liquid. Therefore, such expressions cannot provide an accurate correlation of the system presented in this work, which play a major new role in the formation of microbubbles. However, they allow us to make preliminary evaluations of the resonance frequency and show that it is inversely proportional to the microbubble radius. This observation suggests that a reduction in microbubble size consequently increases the resonance frequency of the microbubble.

### 4.3. Computational Results

A structured mesh consisting of the two inlet channels that pump the liquid and gas into the junction and a stainless-steel outlet capillary where the formed bubbles traverse was used. The fluids in both phases are Newtonian and incompressible. The outlet capillary is polarised with a specific electric field. The geometrical configurations of the T-junction conform to the inner channel diameters of the coaxially aligned capillaries.

The walls were set to a no-slip condition, as the velocity at the walls is zero, the electric field is applied along the walls of the outlet capillary, and the gas and liquid flow rates were set to the values used in the experiments. The conservation equations were solved in a fully implicit manner by means of the Crank–Nicholson method. This, together with the discretisation and interpolation schemes used (central differencing), ensure second-order accuracy in space and time. An auto-time step is selected with a fixed yet conservative Courant–Fredrichs–Lewy (CFL) [48,49] number of 0.5, which regulates the propagation of the fraction of the cell occupied by the fluid within one time-step and ensures that results display minimal dependence on the temporal resolution. An algebraic multigrid method (AMG) was used to iteratively solve the series of algebraic equations [50].

The fluids used for the liquid and gas phase are Bovine Serum Albumin (BSA) and nitrogen, respectively, and their physical and electrical properties were specified in the volume conditions of the CFD-ACE+ solver. The effect of AC on microbubble diameter was not modelled as part of this study.

### 4.4. Formation of Microbubbles

**Figure 5:**
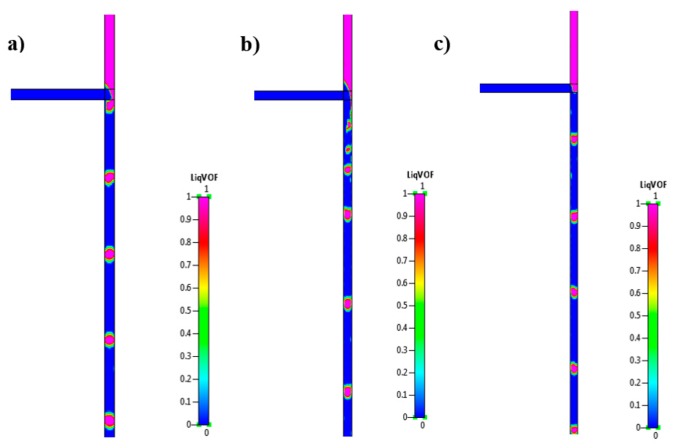
Microbubble formation in the T-junction (**a**) without an electric field; (**b**) DC electric field of 6 kV DC; and (**c**) AC onto DC of 2 kVP–P, 6 kV, 2 kHz.

Three main tests were conducted in this section, firstly to simulate the T-junction without the application of an electric field, followed by the application of a DC field along the capillary outlet walls, and finally superimposing an AC onto a DC electric field. The formation of microbubbles under these conditions is shown in Figure 5.

From Figure 5a, it can be observed that as the gas enters the main fluid channel, it begins to grow until it breaks off, forming a bubble. These bubbles eventually propagate downstream in a well-organised and highly repeatable sequence. Applying a DC field of 6 kV along the walls of the outlet capillary result in smaller bubbles, however, an increase in their polydispersity occurs as shown in Figure 5b. The gas stream is observed to traverse further into the outlet channel before retracting back towards the junction. Introducing an oscillating field results in a steady stream of smaller bubbles that are homogenous in nature (Figure 5c). This steady formation can be attributed to the fact that the application of a frequency generates a regular axisymmetric disturbance at the liquid interface. Increasing the frequency from 0 kHz to 5 kHz results in the decrease in microbubble diameter from 115 µm to 60 µm. These animations are included in SI 6. Utilising the same parameters, the experimental results resulted in a decrease in microbubble diameter from 111 ± 5.3 µm to 31 ± 2.1 µm.

**Figure 6:**
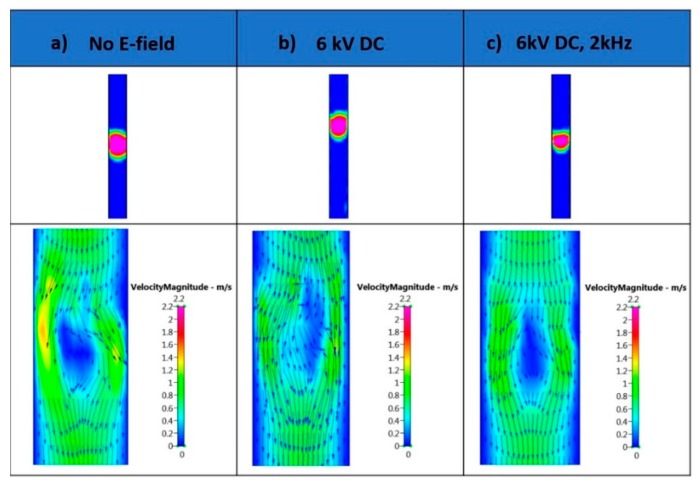
Velocity magnitude vector plots of the microbubble with (**a**) no electric field; (**b**) 6 kV DC; and (**c**) 2 kV P–P AC, 6 kV DC, 2 kHz.

Figure 6a displays the velocity vectors around the bubble without an application of an electric field and under the influence of electrical load. In the absence of an electric field, the velocity vector distributions display slight perturbations as the bubble traverses along the outlet channel, and form microbubbles of 115 µm. Similar results were observed in Figure 6b upon application of a DC electric field, where the bubble diameter was recorded as 108 µm. On the other hand, in the presence of a superimposed field, there is a uniform velocity field distribution along the bubble, as shown in Figure 6c. This contributes to the controlled detachment of the emerging microbubble.

### 4.5. Microbubble Detachment

**Figure 7:**
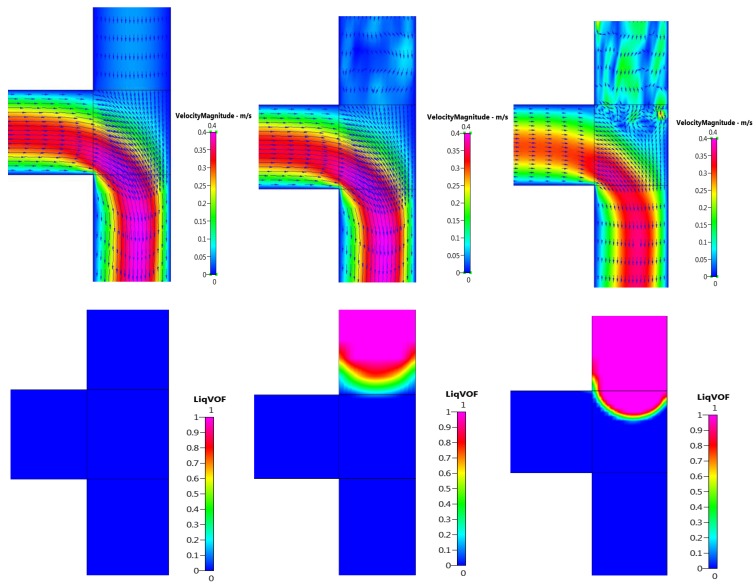
Velocity magnitude plots corresponding with various stages of gas column approaching the mixing region without application of the electric field.

**Figure 8:**
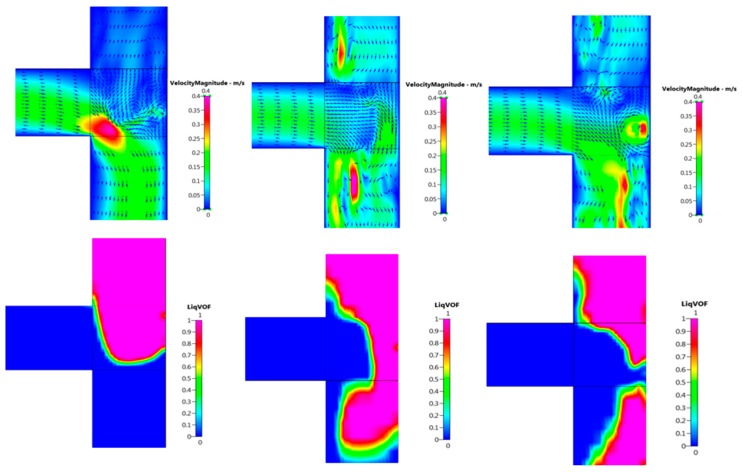
Velocity magnitude contours corresponding to the volume of fluid (VOF) simulation results displaying the bubble detachment and gas column retracting back into the inlet.

Flow in the microchannels is characterised by low Reynolds numbers, as the flow is dominated by viscous stresses and surface tension forces (Re < 100, based on pipe diameter and bulk velocity) [51]. Figure 7 presents images of the gas column approaching the mixing region and the corresponding velocity magnitude vector fields observed at the mixing zone. Just before the gas column enters the mixing region, recirculation is observed possibly as a result of the disturbance in the laminar flow caused by the two fluid media coming into contact with each other. As the gas column enters the mixing region, the vortex cuts through the gas stream and pushes the gas–liquid interface towards the edge of the junction until the neck breaks and forms a bubble. This phenomenon is shown in Figure 8.

Similar flow dynamics was observed by Guo et al. [52] in their numerical investigations on Taylor bubble formation in a microchannel using the VOF method. These authors attributed this circulation as a feature of the ‘squeezing regime’, which has been reported in great detail by De Menech et al. [30]. Past the squeezing stage, the diameter decreases rapidly and the liquid rushes to fill the volume around the neck, as shown by the velocity vectors in Figure 8, showing good agreement with the research conducted by van Steijn et al. [53]. From the velocity plots, it can be seen that as the gas column occupies the junction, there is a distinctive drop in velocity upstream, suggesting a build-up of pressure, which facilitates narrowing of the neck. The recirculation observed after the stream breaks up is the result of the recoiling of the gas column and the surface tension pulling the tail of droplet to form the equilibrium shape [54,55]. Thus, it can be elucidated that the vortex observed in the simulation results contribute to the bubble detachment process.

### 4.6. Comparison between Simulation Results and Optical Micrographs

Microbubbles formed in the simulations and micrographs were scaled and measured using ImageJ and compared with the optical micrographs obtained in the experiments. Smaller bubbles have higher internal pressure and release gas to dissolve under pressure into the surrounding under-saturated solution, whereas larger bubbles grow by taking up gas from a supersaturated solution. The underlying reason behind this phenomenon is that smaller microbubbles shrink as a result of their high Laplace pressure, and thus create areas of supersaturation in the local environment. This causes larger bubbles to grow via a process called Ostwald Ripening [56]. Elevated levels of pressure were recorded in the smaller microbubbles generated using a superimposed AC on DC electric field, as opposed to the larger microbubbles produced in the absence of any electric fields. It should be noted that the Ostwald Ripening process is not modelled in our simulations.

**Figure 9:**
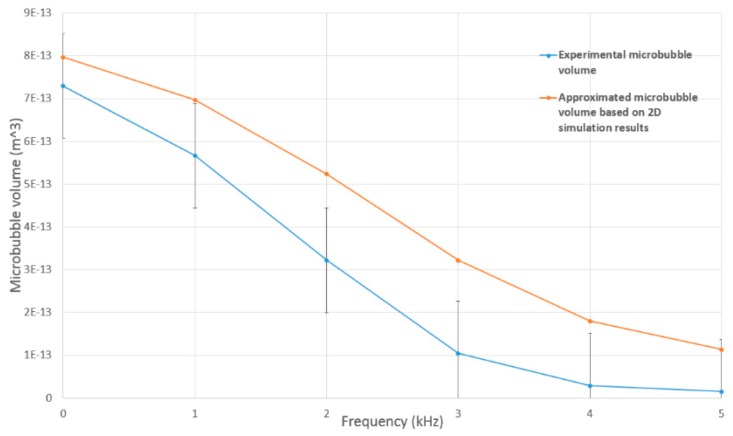
Comparison of calculated microbubble volume from experiments and approximated microbubble volume based on the 2D simulation results at 6 kV DC, 2 kV P–P between 1–5 kHz.

Figure 9 presents a graph of the volume of the microbubbles obtained via the experiments against the approximated volume from the 2D simulation results. A good trend between the results can be seen, the discrepancy between the two curves attributes from the uncertainty in the VOF boundary condition, as only 2D simulations were computed as part of this work. This also suggests the need for a finer grid in order to resolve the boundaries more accurately.

## 5. Conclusions

A novel experimental set-up was developed to superimpose an AC electric field onto a DC electric field. This introduces new variables such as applied AC voltage and frequency, which were investigated in detail. The experimental set-up allows the superimposition of an AC onto DC by adjusting the offset on the waveform generator. It was observed that increasing the applied AC results in the formation of larger microbubbles, possibly as a result of consequent heating effects. On the other hand, increasing the frequency resulted in a decrease in microbubble diameter. The distinctive drop in bubble diameter observed between 600–900 Hz can possibly be attributed to the resonance of a bubble of that diameter because of the applied frequency, which is suggested by Minnaert’s theory for resonance. In the numerical simulations of the microbubble formation, disturbances in the flow field were observed in the microbubbles formed in the absence of an electric field. On the other hand, we recorded a steady flow field along the bubble interface when a superimposed field was applied in combination with the formation of smaller microbubbles. Recirculation before the gas column enters the mixing region is possibly the result of the disruption in the laminar flow caused by the two-fluid media coming into contact at the junction. As the gas column enters the mixing region, the liquid stream cuts through the vortex and pushes the gas–liquid interface towards the edge of the junction until the neck breaks and forms a bubble, known as the ‘squeezing regime’. The vortex behaviour recorded in the simulation results contributes to the bubble detachment process. The experimental results acquired suggest that the bubble size can potentially be further reduced by utilising higher frequencies, allowing better control over the microbubble size. Further experimentation will be conducted in order to grasp a deeper understanding of the underlying physics and further optimise the apparatus; in order to move towards achieving microbubbles in the 2–8 µm range for biomedical applications.

## Figures and Tables

**Table 1:** Characteristic properties of the solution used in the experiment. PEG-40-S—polyethylene glycol-40-stereate.

Solution	Viscosity (mPa·s)	Surface Tension (mN·m^−1^)	Electrical Conductivity (µS·m^−1^)	Density (kg·m^3^)
**50% wt. Glycerol + 1% wt. PEG-40-S**	8	54	2.0	1100

## Supplementary Materials

The following are available online at http://www.mdpi.com/2072-666X/9/10/497/s1, S1: Schematic of pin arrangement, S2: Bubble formation at 2 kV, 10 Hz, S3: Bubble formation at 4 kV, 10 Hz, S4: Bubble formation at 6 kV, 10 Hz, S5: Bubble formation at 8 kV, 10 Hz, S6: animations of bubble formation under different electric field scenarios.
