# Supplementary material for: Novel Preparation of Monodisperse Microbubbles by Integrating Oscillating Electric Fields with Microfluidics
Source: Micromachines (Basel). 2018 Sep 27;9(10):497. doi: 10.3390/mi9100497 (PMC6215214; doi:10.3390/mi9100497)
Supplement: Supplementary file 1 [file micromachines-09-00497-s001.zip › Supplementary Information/SI 1.pdf]

Supplementary Material

SI 1

The High Voltage amplifier (HVA) is used to amplify a low voltage input, it is a crucial component of this experimental set-up. This unit is fitted with a differential operational amplifier. Differential amplifiers generate an amplified output of the difference between two input voltage signals. The positive(+) or live terminal from the voltage source is connected to the positive (+) pin of the Voltage programming pin (V2) and negative(-) pin is connected to ground. Therefore, the output voltage can be expressed as:

$$V_{out} = V_2 - V_1$$

An alligator clip was soldered to the output cable of the HVA which was fastened securely to the stainless steel capillary. An 'O' connector was soldered to a wire, which screwed on to the High Voltage return and connected to the platform to complete the circuit. The parameters being tested were set on the Frequency Generator, and the low voltage power supply enabled the HVA to power-up. Prior to any experiments, the FEP capillaries and the stainless steel capillary were inspected for blockages, to ensure there was no obstruction to flow.

a)

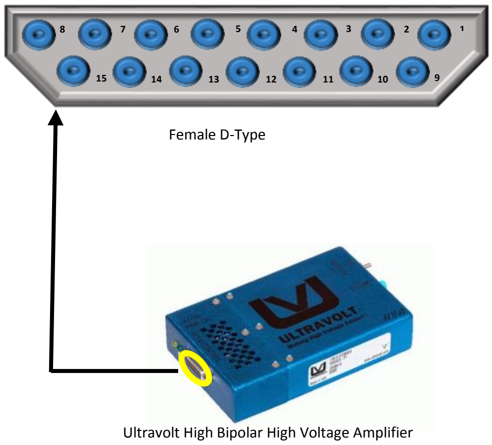

Input Connector Pin Functions	
**Pin**	**Description**
1	+ 10V Reference voltage
2	Voltage Programming (-)
3	Voltage Programming (+)
4	Voltage Monitor
5	N/C
6	Signal Ground
7	Input Power
8	Input power
9	Power Ground
10	Power Ground
11	Enable
12	Current Monitor
13	Current Limit Adjust
14	N/C
15	Signal Ground

b)

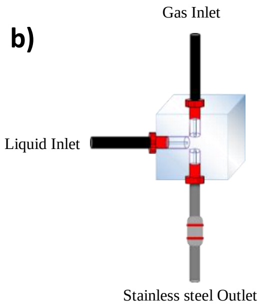

Calibration of High Voltage Amplifier

In order to verify that the voltage being supplied by the HVA is the same as the one being supplied to the load, a High Voltage Probe (Fluke,80k-40) embedded with 10MΩ resistor which acts as a 1000:1 potential divider. It was connected to a digital multimeter (Fluke, 10A ac, 1000V AC). The high impedance HV probe essentially steps-down the measured voltage by a factor of 1000V, i.e. a measurement of 2kV was recorded as 2V on the multimeter. The AC component was also checked, by changing the setting on the multimeter. It was recorded as an rms voltage, this was converted into a P-P voltage by(Association):

$$V_{rms} = \frac{V_0}{\sqrt{2}}$$

Calibration of Input and Output Signals

The signal that drives the inputs of the HVA was verified against the output signal from the HVA. A direct connection from the waveform generator was made to channel 1, using a BNC T-connector piece, from which the second connection was made to the input of the HVA. A direct connection was made from the monitoring pin on the HVA, to view the output signal. The signals were relatively in phase, apart from a slightly distorted output signal due to noise prevalent in the amplifier, which generates a slight harmonic distortion(Rijns, 1996).

References

ASSOCIATION, P. C. A. R. Peak-to-Peak, RMS Voltage, and Power. 1732 Forest Circle Balsam Lake WI: Polk County Amateur Radio Association.

RIJNS, J. 1996. CMOS low-distortion high-frequency variable-gain amplifier. *Solid-State Circuits, IEEE Journal of,* 31**,** 1029-1034.
