# Supplementary material for: Novel Preparation of Monodisperse Microbubbles by Integrating Oscillating Electric Fields with Microfluidics
Source: Micromachines (Basel). 2018 Sep 27;9(10):497. doi: 10.3390/mi9100497 (PMC6215214; doi:10.3390/mi9100497)
Supplement: Supplementary file 1 [file micromachines-09-00497-s001.zip › Supplementary Information/SI 6.pptx]

## Slide 1
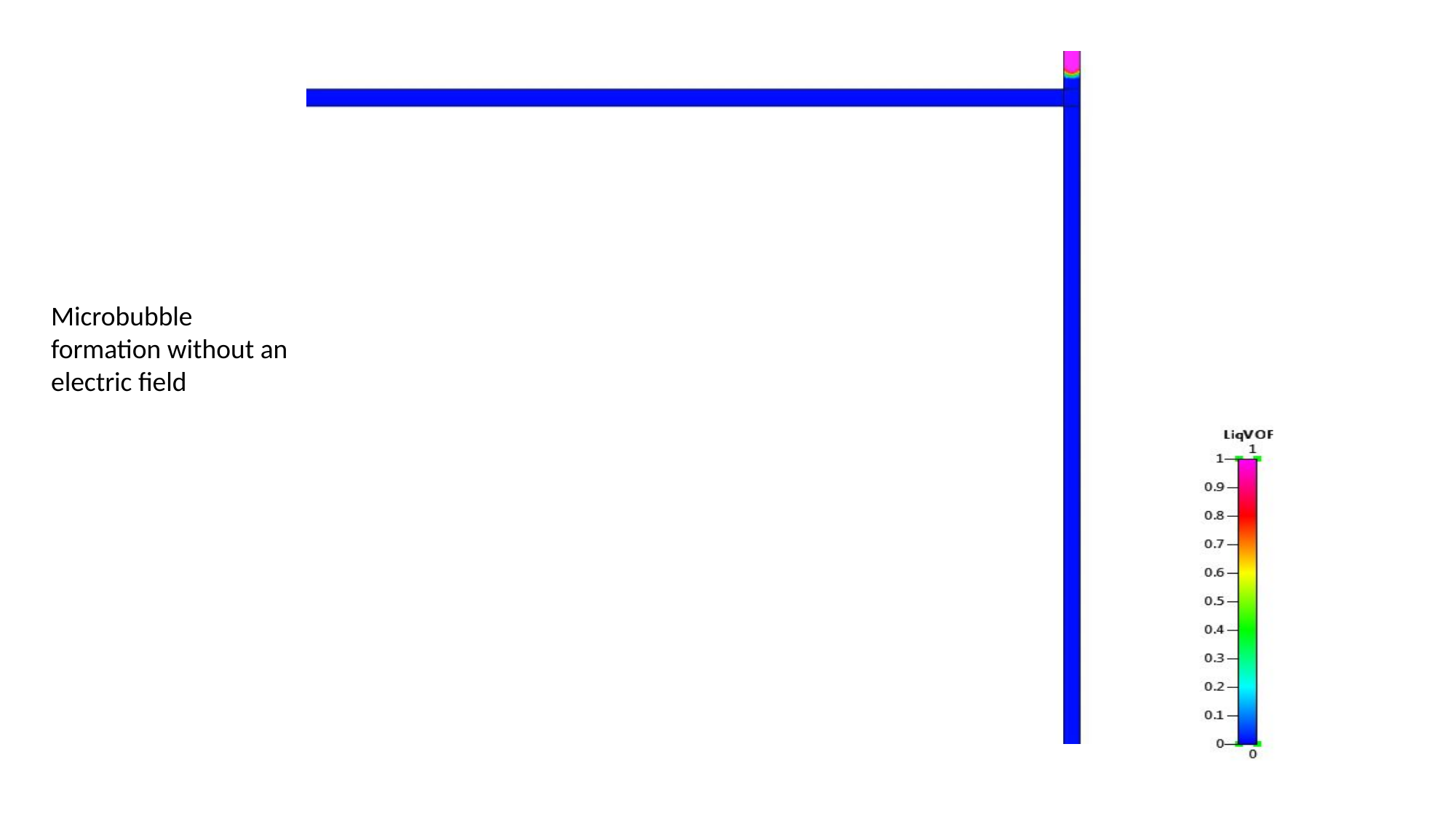

Microbubble formation without an electric field

## Slide 2
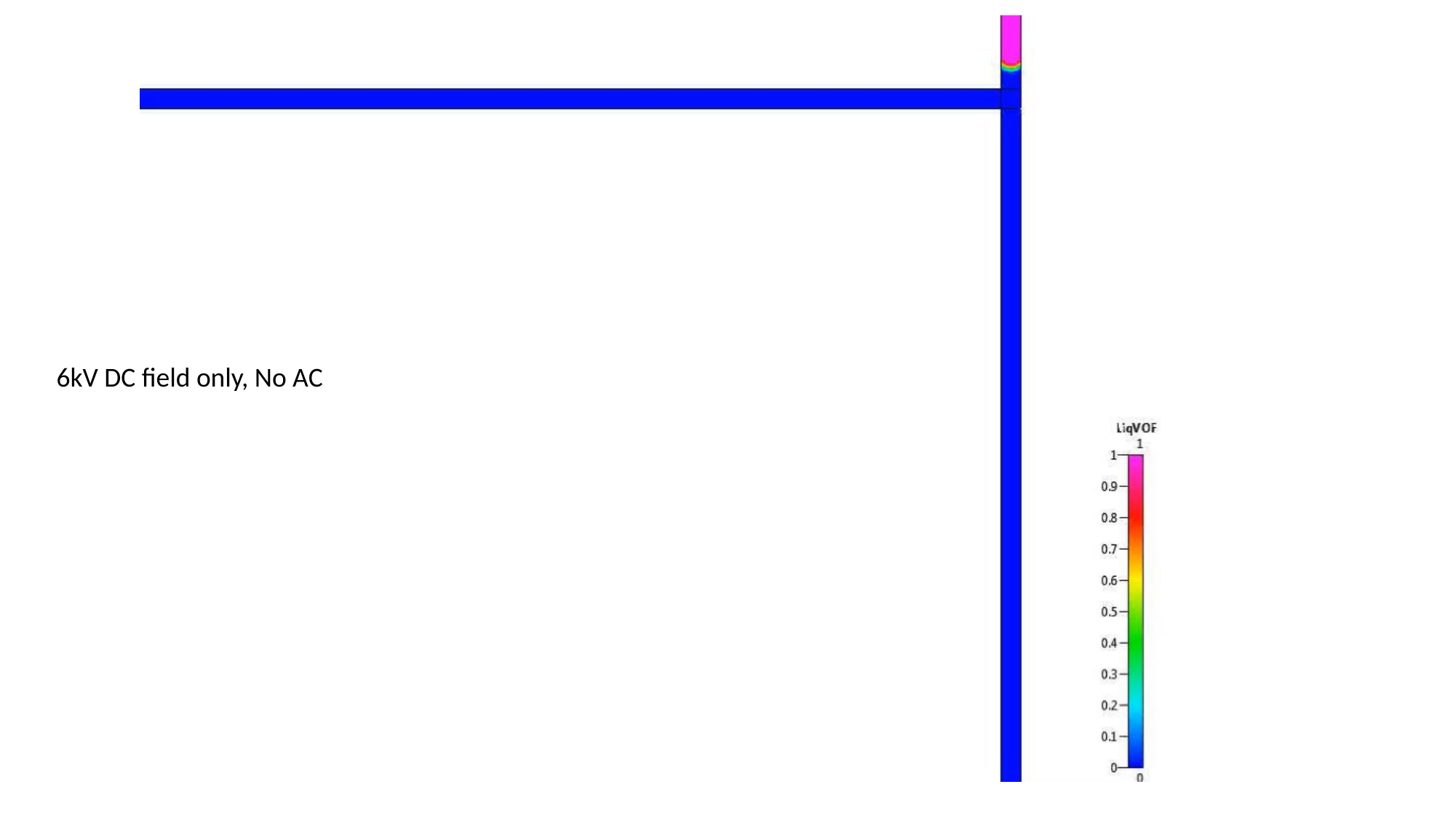

6kV DC field only, No AC

## Slide 3
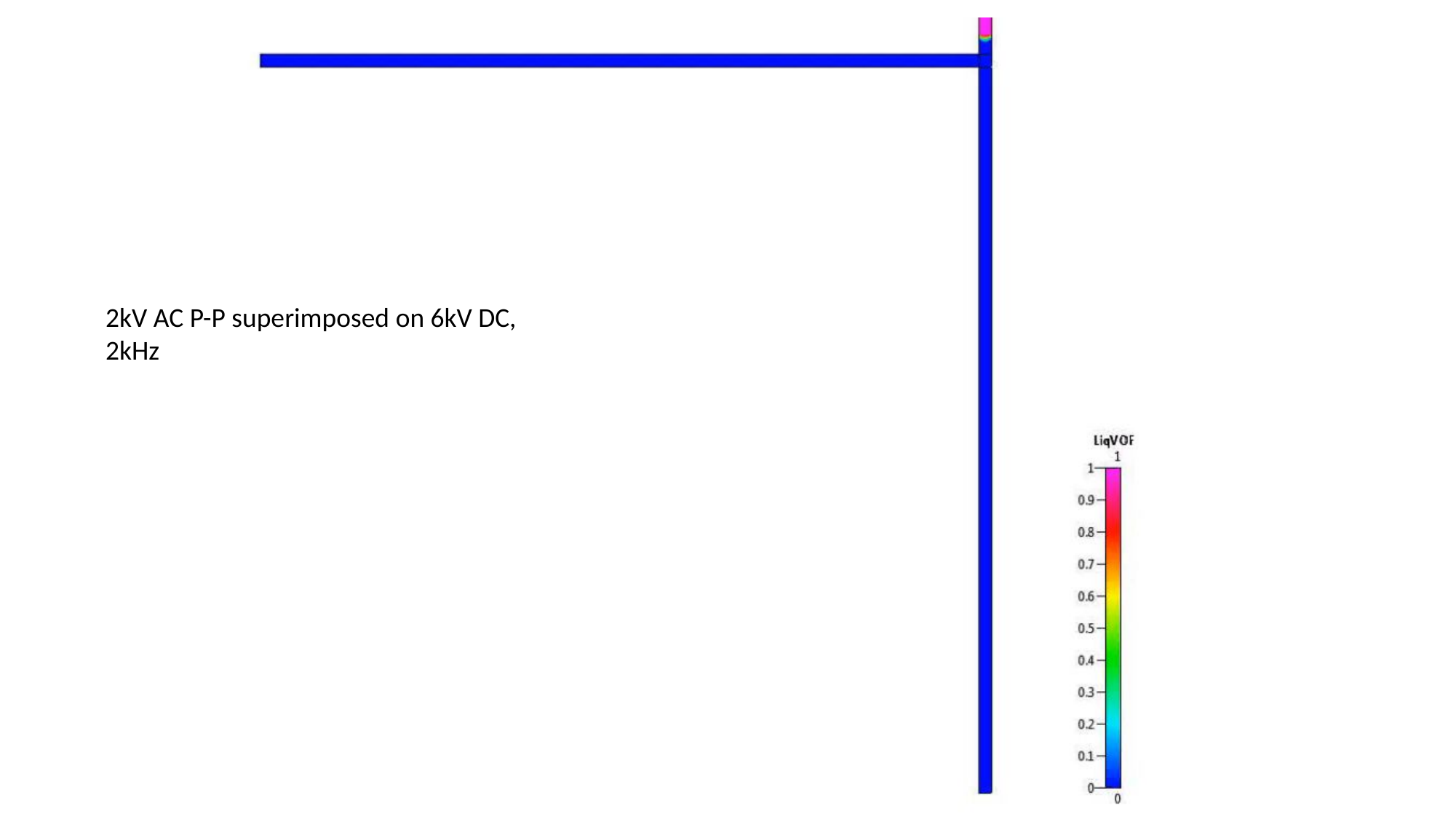

2kV AC P-P superimposed on 6kV DC, 2kHz
